# Sonoelastography in differentiating benign and malignant thyroid nodules: A histopathological correlation

**DOI:** 10.6026/973206300213744

**Published:** 2025-10-31

**Authors:** Md Shahrukh Ansari, Suresh Kumar Toppo, Rajeev Kumar Ranjan, Anima Ranjni Xalxo, Sonali Priyadarsini Reddy, Soumik Pal, Riya Agarwal, Sumaiya Shikoh

**Affiliations:** 1 Department of Radio-diagnosis, Rajendra Institute of Medical Sciences, Ranchi, Jharkhand, India; 2 Department of Obstetrics and Gynaecology, Tata main Hospital, Kadma, Jamshedpur, Jharkhand, India

**Keywords:** Thyroid nodules, sonoelastography, ARFI, ultrasound, malignancy, histopathology

## Abstract

Thyroid nodules are common and distinguishing benign from malignant ones is essential for optimal management. This cross-sectional study of 120 patients assessed Acoustic Radiation Force Impulse (ARFI) imaging in differentiating thyroid nodules, correlating results with histopathology. Malignant nodules showed higher mean ARFI values (3.59 ± 0.7 m/s) than benign ones (1.98 ± 0.31 m/s). With a 2.8 m/s cutoff, ARFI achieved 92.6% sensitivity and 92.9% specificity. Combining ARFI with conventional ultrasound improved diagnostic accuracy, highlighting its potential to reduce unnecessary biopsies.

## Background:

Thyroid nodules are a common clinical finding, affecting approximately one-third of adults between 18 and 65 years of age, with even higher prevalence in iodine-deficient regions [1]. While the majority of thyroid nodules are benign, approximately 5-15% harbor malignancy, necessitating accurate differentiation to guide appropriate management [2]. The challenge lies in reliably distinguishing benign nodules from malignant ones without subjecting patients to unnecessary invasive procedures. Physical examination has limited accuracy in assessing thyroid nodules, particularly for multiple, small, or deeply located nodules [3]. Conventional ultrasound (US) is the primary imaging modality for evaluating thyroid nodules, providing detailed anatomical information. Certain US characteristics, such as marked hypo-echogenicity, irregular or spiculated margins, microcalcifications and a taller-than-wide shape, are associated with malignancy [4]. However, the interpretation of these features is subject to significant inter-observer variability [5, 6]. Fine-needle aspiration biopsy (FNAB) is the cornerstone of thyroid nodule evaluation, with high specificity (60-98%) but variable sensitivity (54-90%) for detecting malignancy [7]. This limitation results in many patients with benign thyroid nodules undergoing unnecessary invasive procedures. Current guidelines recommend FNAB for nodules ≥10 mm in patients with normal thyroid-stimulating hormone (TSH) levels and for smaller nodules with suspicious US features or concerning clinical history [8]. Elastography is an emerging ultrasound-based technique that evaluates tissue stiffness, which often correlates with pathological changes. Malignant thyroid nodules tend to be harder than benign ones due to increased cellularity, desmoplastic reactions and calcifications [9]. Among various elastography techniques, Acoustic Radiation Force Impulse (ARFI) imaging has gained attention for its quantitative assessment of tissue stiffness, operator independence and reproducibility [10, 11]. Recent studies have demonstrated the potential of ARFI in differentiating thyroid nodules. Therefore, it is of interest to evaluate the diagnostic efficacy of ARFI elastography in differentiating benign from malignant thyroid nodules and to correlate its findings with histopathological results.

## Materials and Methods:

## Study design and setting:

This was a hospital-based cross-sectional study conducted at the Department of Radiodiagnosis, Rajendra Institute of Medical Sciences (RIMS), Ranchi, between April 2024 and January 2025. Ethical clearance was obtained from the Institutional Ethics Committee (IEC) with memo no. 127 dated 06/04/2024.

## Sample size:

A total of 120 patients with thyroid nodules were included in the study, comprising 37 males and 83 females aged between 18 and 65 years.

## Inclusion criteria:

Patients were included if they had:

[1] One or more solid thyroid nodules larger than 5mm

[2] Mixed (solid and cystic) nodules with a solid component larger than 5mm

[3] Normal levels of thyroid-stimulating hormone (TSH)

[4] Provided written consent for tissue sampling/resection

## Exclusion criteria:

Patients were excluded if they had:

[1] Purely cystic nodules or mixed nodules with a solid component smaller than 5mm

[2] Ongoing diffuse thyroiditis

[3] No tissue samples available for histopathological analysis

[4] Indeterminate biopsy results without repeat procedure

## Data collection:

Detailed demographic and clinical data were recorded for each patient, including age, gender, symptoms, family history of malignancy and history of radiation exposure. All patients provided written informed consent before undergoing any procedures.

## Conventional ultrasound examination:

Patients were positioned supine with the neck extended. Conventional B-mode ultrasound was performed followed by color Doppler using a Philips HEUS Affinity-70G ultrasound machine. Nodules were evaluated for:

[1] Composition: Solid or mixed

[2] Echogenicity: Hyperechoic, isoechoic, hypoechoic, or markedly hypoechoic

[3] Margins: Smooth, microlobulated, or irregular

[4] Calcifications: Microcalcifications (≤1 mm) or macrocalcifications (>1 mm)

[5] Shape: Taller-than-wide or wider-than-tall

[6] Vascularity on color Doppler: No vascularity, peripheral, intra-nodular, or both

Nodules were classified based on the presence of suspicious features (marked hypo-echogenicity, microcalcifications, irregular margins, intra-nodular vascularity, or taller-than-wide shape) into:

[1] Benign

[2] Probably benign

[3] Suspicious for malignancy

[4] Highly suspicious for malignancy

## ARFI imaging:

ARFI imaging (Virtual Touch Tissue Quantification, VTQ) was performed using the same Philips HEUS Affinity-70G ultrasound machine with a 4-18MHz probe. The region of interest (ROI) was set to 1 cm x 0.5 cm and placed to cover the entire nodule, avoiding areas of degeneration, fluid, blood vessels, calcifications, or scar tissue. For larger nodules, multiple measurements were taken at different locations and the average ARFI value was calculated. In patients with multiple nodules, ARFI was performed on the most suspicious nodule or the largest one if none appeared suspicious. Four measurements were taken for each nodule and two for the surrounding thyroid tissue (2-3 cm from the nodule, avoiding blood vessels). Measurements were performed during brief breath-hold. Results expressed as "x.xx m/s" were excluded from the analysis. Shear wave velocity was expressed in meters per second (m/s).

## Histopathological examination:

All patients underwent either fine-needle aspiration biopsy (FNAB) and/or surgical resection for histopathological examination. Two experienced pathologists examined all tissue samples to establish the final diagnosis.

## Statistical analysis:

Data were analyzed using SPSS 11.0 and Systat 8.0 software. Microsoft Word and Excel were used to create graphs and tables. Descriptive statistics were calculated for all variables. The diagnostic performance of conventional ultrasound and ARFI was evaluated by calculating sensitivity, specificity, positive predictive value (PPV), negative predictive value (NPV) and accuracy. The chi-square test was used to compare categorical variables and the t-test was used for continuous variables. A p-value < 0.05 was considered statistically significant.

## Results:

Table 1 (see PDF) summarizes the demographic and clinical characteristics of the study population. Patients with malignant nodules were significantly older (mean age 53.83 years) than those with benign nodules (44.68 years, p < 0.05). Radiation exposure was strongly associated with malignancy (58.3% vs. 18.5%, p < 0.05), while visible neck swelling was exclusively observed in benign cases (100% benign, 0% malignant, p < 0.05). Family history of malignancy showed a non-significant trend toward higher malignancy rates (35% vs. 20%, p = 0.28). Table 2 (see PDF) details ultrasound characteristics predictive of malignancy. Hypoechoic nodules were predominantly malignant (66.7%, p < 0.05), while hyper-echoic nodules were exclusively benign. Irregular margins (68.2% malignant) and microlobulated margins (100% malignant) were significant malignancy indicators (p < 0.05). Microcalcifications were pathognomonic for malignancy (100%, p < 0.05). Ultrasound alone demonstrated high specificity (94.9%) but moderate sensitivity (56.1%). Table 3 (see PDF) presents ARFI elastography findings. Malignant nodules exhibited significantly higher mean ARFI velocities (3.59 m/s) than benign nodules (1.98 m/s, p < 0.05). Among specific lesions, follicular carcinoma showed the highest ARFI value (4.9 m/s). Using a 2.8 m/s cutoff, ARFI achieved 91% sensitivity and 97.3% specificity, with 95.8% accuracy in distinguishing malignant nodules. Table 4 (see PDF compares diagnostic performance across modalities. ARFI alone (2.8 m/s cutoff) outperformed ultrasound in sensitivity (92.6% vs. 56.1%) and NPV (97.8% vs. 80.6%). The combined ultrasound-ARFI approach improved sensitivity to 74.35% while maintaining high specificity (93.9%), offering the most balanced diagnostic profile.

## Discussion:

The demographic analysis revealed that malignancy rates increased with age, reaching 50% in patients above 60 years, though this correlation was not statistically significant. This finding is consistent with previous studies that have reported an increased risk of thyroid malignancy with advancing age [12, 13, 14-15]. Gender distribution showed a higher malignancy rate in females (30.1%) compared to males (5.4%), which aligns with the known higher prevalence of thyroid nodules and cancer in women [16]. Notably, patients with a history of radiation exposure had a significantly higher malignancy rate of 58.3%, confirming radiation as a strong risk factor for thyroid cancer [17, 18]. Interestingly, all cases with visible neck swelling were benign, while malignancy was only found in cases without visible swelling. This observation may be related to the fact that benign nodules often grow larger and become more palpable, while malignant nodules may remain impalpable despite their aggressive nature [19]. Conventional ultrasound findings in our study showed that hypoechoic nodules were more frequently malignant (66.7%), whereas hyper-echoic nodules were exclusively benign. These findings are consistent with previous reports that have identified hypo-echogenicity as a significant predictor of malignancy [20, 21]. Nodule margin analysis further demonstrated that malignancy was strongly associated with irregular or microlobulated margins, while benign nodules predominantly had regular margins, supporting previous observations [22, 23]. The presence of microcalcifications was a highly significant predictor of malignancy in our study, as all nodules with microcalcifications were malignant. This finding is consistent with numerous previous studies that have identified microcalcifications as one of the most specific US features for thyroid malignancy [24, 25]. Color Doppler findings revealed that intra-nodular vascularity was present in 58.6% of malignant cases, whereas peripheral vascularity was exclusively observed in benign nodules, supporting the utility of vascularity patterns in thyroid nodule evaluation [26, 27]. Despite these well-established US features, our study confirmed the limitations of conventional ultrasound in thyroid nodule evaluation. The sensitivity of ultrasound alone was only 56.1%, meaning that nearly half of malignant nodules were not correctly identified. This limitation has been recognized in previous studies and highlights the need for complementary diagnostic tools [28, 29]. ARFI elastography proved to be a highly effective tool for malignancy prediction in our study. Nodules with an ARFI value >2.8 m/s had a malignancy rate of 92.6% and the mean ARFI value for malignant nodules (3.59 m/s) was significantly higher than that for benign nodules (1.98 m/s). These findings are consistent with previous studies that have reported higher ARFI values in malignant thyroid nodules [30, 31, 32, 34- 35]. Our results, with mean ARFI values of 1.98 ± 0.31 m/s for benign nodules and 3.59 ± 0.7 m/s for malignant nodules, are consistent with these previous reports. Analysis of specific thyroid lesions in our study showed that benign conditions such as adenomas and colloid nodules had lower mean ARFI values (2.03 and 1.9 m/s, respectively), whereas malignant conditions like papillary carcinoma, medullary carcinoma, lymphoma and follicular carcinoma exhibited much higher ARFI values (3.4, 3.3, 3.97 and 4.9 m/s, respectively). The optimal ARFI cutoff value for differentiating benign and malignant thyroid nodules has varied across studies. Chung *et al.* suggested an ARFI cutoff of 2.57 m/s [12], in our study, an ARFI cutoff of 2.8 m/s provided the best balance of sensitivity (91%), specificity (97.3%) and accuracy (95.8%). Using this cutoff value, an ARFI showed high sensitivity (92.6%), specificity (92.9%), PPV (78.1%) and NPV (97.8%) in our study. Our study has several limitations. First, values noted as "X.XX m/s" was excluded, as these were usually found in very hard nodules. Second, we measured the ARFI velocity only in the solid portions of the nodule, avoiding areas with fluid, blood vessels, or scar tissue, which might have affected the results. Third, our study involved 120 nodules in 120 patients and larger studies may be helpful in confirming our results. Finally, we had only a small number of each type of cancer, so we couldn't compare ARFI values between different types of malignant tumors. Despite these limitations, our study supports the value of ARFI elastography in the evaluation of thyroid nodules. ARFI provides additional information about tissue elasticity that complements the anatomical information provided by conventional ultrasound. The combination of ARFI with conventional ultrasound improves diagnostic accuracy and may help reduce unnecessary biopsies while ensuring timely detection of malignant nodules. Generally, sonoelastography serves as a valuable, noninvasive diagnostic adjunct in evaluating thyroid nodules, helping clinicians distinguish benign from malignant lesions based on tissue stiffness characteristics. It enhances the diagnostic accuracy of conventional ultrasound, potentially reducing the need for unnecessary biopsies. However, its effectiveness depends on operator skill and standardized scoring systems, and larger studies are needed to validate its clinical utility and establish uniform diagnostic criteria [36].

## Conclusion:

We show that ARFI elastography is a valuable tool for differentiating between benign and malignant thyroid nodules. Malignant nodules had significantly higher mean ARFI values (3.59 ± 0.7 m/s) compared to benign nodules (1.98 ± 0.31 m/s). Using an ARFI cutoff value of 2.8 m/s, the technique showed high sensitivity (92.6%) and specificity (92.9%) for detecting malignancy. Conventional ultrasound features associated with malignancy included hypo-echogenicity, irregular margins, microcalcifications and intra-nodular vascularity.
